# Between Stigmatization and Acceptance: Diabetic Patients as Civil Servants in West Germany, 1950–1970

**DOI:** 10.1007/s00048-022-00325-y

**Published:** 2022-02-15

**Authors:** Livia Prüll

**Affiliations:** Universitätsmedizin der Johannes Gutenberg-Universität Mainz, Mainz, Germany

**Keywords:** Diabetes, Civil Servant Employment, Transfer of Medical Knowledge, History of West Germany, Patient History, Diabetes, Verbeamtung, Medizinischer Wissenstransfer, Geschichte Westdeutschlands, Patientengeschichte

## Abstract

Patient history has enriched medical history since about the 1980s. But there are still research gaps in certain periods and themes, especially in topics related to the medical history of West Germany. This paper deals with the efforts of patients, lay persons, and medical advisors (diabetologists) to enable diabetics to secure employment as civil servants (*Verbeamtung*). Attention will be payed to the fact that this success relied on the activities of mediators, who translated and conveyed the patients’ interests to society at large. This victory was concordant with similar initiatives in other fields of the diabetic life, including sexuality and lifestyle management. Therefore, efforts to achieve civil servant employment for diabetic patients were constitutive of a broader initiative that changed the image of the disease and promoted the integration of diabetic patients into West German society.

## Abstract (de)

Wiewohl die Patientengeschichte seit Mitte der 1980er Jahre die Medizingeschichte stark bereichert hat, finden sich noch starke Forschungsdesiderate, so beispielsweise im Rahmen der Forschung zur Medizingeschichte der frühen Bundesrepublik. Der Beitrag behandelt die Bemühungen von Diabeteskranken auf der einen Seite sowie Laienhelfern und Mediziner_innen, d. h. Diabetologen, auf der anderen Seite, um einen Zugang zur Verbeamtung von Patient_innen. Es wird herausgearbeitet, dass die Erfolge auf der Arbeit von Mediatoren beruhte, welche die Bedürfnisse der Diabeteskranken mit der Gesellschaft aushandelten. Diese Erfolge gingen einher mit ähnlichen Bemühungen auf anderen Gebieten der diabetischen Lebensführung (Sexualität, Diskussionen zum Lebenssinn des Diabeteskranken). Damit trug der Einsatz für die Verbeamtung von Diabeteskranken dazu bei, das Image der Krankheit zu verändern und eine Integration in die westdeutsche Gesellschaft zu fördern.

## Diabetic Patients and Their Vision of Life

Since about 1985, when the path-braking paper of Roy Porter was published, teaching and research on the history of the patient attracted increasing attention. This approach to patient history was very much embedded in cultural history; it involved examining the dependency of diagnostics and treatment on national habits and attitudes. Researchers focused mainly on low status social groups who stood at the margins of society and favored alternative healing systems (Porter 1985; Loetz 1993). This paper deals with transfer processes and decision-making between medical healers and patient groups whose representatives did not solely see themselves as ardent defenders of their own medical theories and systems operating in a restricted world of diagnostic and therapeutic methods. In contrast, these social groups viewed their debates with physicians as a defense of their own way of life, they believed that there was much more at stake than the choice of appropriate medical tools and methods. These patient groups even questioned their own status as bearers of a pathological condition, claiming their own right to define their identity and to position themselves within society.

Especially when looking at these marginalized patient groups, one still comes across significant research gaps, especially with respect to those historical periods and locations which have generally received little attention.^1^ One important piece for understanding the attitude of these groups is coming to understand the origins of their self-estimation, self-confidence and their wishes for self-help. Did these arise as the result of the delegation of responsibity from doctors, who generally enjoyed a monopoly on disease interpretation? Yet many of these doctors had also used patient collaboration as a tool to solve medical problems and therefore loosened ties of patient control. Over the last forty years, since the uptake of research in social history of medicine, historians have come to see how decision-making in this area was maintained within networks on the basis of knowledge transfer processes, ideas, and visions of quality of life. Crucially, it was not solely based on topdown medical action and impulses (Wolf 1998; Eckart & Jütte 2007, 181–190)^2^. In contrast, patients themselves could play a determining role in the vacuum left by reduced medical control. Sometimes, they were compelled to do this in order to substantially improve their living conditions.^3^

In the following, we will explain these circumstances using the example of diabetic patients. This group is exemplary due to its long-lasting history of empowerment. By tracing the group’s activities in West Germany, we can examine the interrelations of time-bound social affairs, medical problems and the development of group consciousness. In spite of the surge in research interest on medicine in the early Federal Republic after the collapse of Nazi Germany, with its focus on the mode and strategies of the reinstatement of the physician-patient relationship after 1945, the field demands further attention (see e.g. Oehler-Klein & Roelcke 2007; Stoll 2017; Geisthövel & Hitzer 2018). The stories of diabetic patients and their advocacy is a promising topic. Ylva Söderfeldt demonstrated this on the regional level when describing the self-organization and self-help in the German state of Saarland between 1950 and 1970(Söderfedt 2020: 30–42). This study adopts the same timeframe and pursues one specific example of the fate of diabetics in the Federal Republic in greater detail.^4^ This is the story of how diabetics, a stigmatized patient group, were successful in translating the meaning of their disease in the field of occupational politics and employment law. This success was only possible on the basis of a “translation” of their interests by lay persons as well as diabetes specialists. After the defeat of Nazi Germany, the history of the western part of the country was very much shaped by reconstruction and by the establishment of democracy with the consolidation of the new political system until about the 1970s (Kleßmann 1991; Schildt 2001; Kersting et al. 2010; Gerst 1997).

This article examines to what degree diabetic patients were successful in obtaining access to the position of civil or public servants (*Beamte, Verbeamtung*) in the period from 1950 to approximately 1970. The paper wants to elucidate how patients, lay-people and therapists translated a new concept of the disease of diabetes for an audience of politicians. This translation was necessary not only to achieve acceptance and tolerance in general, but also for securing employment in post-war Germany. This engagement was shaped by the socio-political conditions in West Germany after 1945, since the process of democratization corresponded with an increasingly influential media landscape in which the specific interests of social groups could be articulated (Bösch & Frei 2006; Sammer 2015). Medical practitioners were particularly sensitive to their public appearance, as they attempted to restore a reputation damaged by their political engagement before 1945 (Oehler-Klein & Roelcke 2007; Oehler-Klein 2007; Seemann 2002; Prüll 2010: 107–110).

The basis of this enquiry is a hermeneutic analysis of printed sources and especially the journal *The Diabetic* (*Der Diabetiker*) as the organ of the self-help society The German Diabetic Association (*Deutscher Diabetiker Bund*). Also, unprinted material from different ministries of the Federal Republic will be considered (Labor and Social Organisation, Internal Affairs, Youth, Family and Health and last but not least the Ministry of Health). These files are kept by the German Federal Archive in Koblenz. Based on this material, it is possible to develop a much more detailed description and analysis of the path to civil service than exists in the short comments within the literature to date.

In the following, I will first describe important preconditions of the process while concentrating on diabetes and its history up to 1945. I then deal with the status of diabetic patients in West Germany after 1945 and explore first initiatives by diabetics to gain access to civil servant positions in 1958. In the third subsection, I focus on new discussions that arose in 1969 and led to a more subtle understanding of diabetes and diabetic patients in the political sphere. Finally, in a fourth subsection, I analyze my findings and make some concluding remarks.

## Diabetes and its History

Only in the last decades of the nineteenth century did the signs and symptoms of diabetes become interpreted into modern terms. Physicians located the origins of the disease in the pancreas, which became incapable of producing enough insulin. This condition resulted in problems utilizing glucose and inhibited the processing of fats. Around 1900, a person with diabetes had a life span of approximately 15 years and could expect to die of starvation. The only medical measure available to combat the disease was a strict low-calorie diet with severe restriction of carbohydrates. Managing the disease was only possible on the basis of a close cooperation between patient and physician. This cooperation was embedded in a system of paternalistic medicine where the patient received clear directives from his medical supervisor (Feudtner 1995; Eich 1975: 6–21).

The appearance of diabetes changed decisively in 1921. In this year, the two Canadian researchers Frederick Banting (1891–1941) und Charles Best (1899–1978) successfully extracted and isolated insulin. Although the impact of “medical discoveries” should not be overestimated, there is no doubt that the isolation of insulin changed lives and transformed the social context of diabetic patients tremendously. By substituting insulin, patients could nearly reach the age of physiologically healthy persons. This treatment required careful attention to the regular supposition of insulin and the observance of strict dietary measures. Caring for diabetic patients shifted from accompanying a dying human to accompanying a chronically-ill patient by treating the long-term consequences of her disease—e.g. blood vessel damage or damage to the retina. The shift of treatment and appearance of diabetes began in the 1920s and set off a wave of research interest in diabetes.^5^ Elliot P. Joslin (1869–1962), a US-based pioneer of diabetes treatment since the end of the nineteenth century, introduced modern cooperative diabetes therapy by introducing diabetes counseling by nurses and physicians, as well as organizing vacation camps for children with diabetes. This new approach to therapy influenced many spheres of life, including the labor and job market (Wrenshall & Hetenyi 1962; Bliss 1982; Feudtner 2003). Nevertheless, in Germany, during the “Third Reich,” diabetics had to subordinate their interests to the aims of the national community (*Volksgemeinschaft*). Maintaining individual health was subordinated to contributing to victory at war. Insulin was rationed and hardly available. Diabetic patients in the “Third Reich” were reduced to the old image of the starving patient with a life expectancy of only a few years. This image came to dominate popular attitudes towards diabetics and survived the end of World War II (Roth 1993: 4–9).

## Diabetes Patients After 1945 and Their Access to Civil Servant Positions (1958)

After 1945, amid a surge of new philanthropic ventures, an initiative for diabetes was started by the journalist Robert Beining (1898–1961). Already during the 1930s Beining dealt with problems of nutrition and he became one of the early pioneers of diabetic nutrition in West Germany, promoting this innovative field of interest. In doing so, Beining acted an agent who could bridge the gap between diabetic patients and German society. Together with six other people he founded the German Diabetic Association (*Deutscher Diabetiker Bund, DDB*) in 1951. The aim of this association was to support patients and their families and to advocate for the integration of diabetic patients in West German society.^6^

Also in 1951, Beining and his colleagues founded the journal *The Diabetic* (*Der Diabetiker*) to discuss the health as well as the social problems facing diabetic patients. Very soon thereafter, Beining asked physicians to join the editorial board of the journal. Already in 1951, a medical advisory board (*Beirat*) was founded.^7^ The physicians soon achieved a dominant position in the journal, since they felt responsible for the supervision of diabetic patients and their struggles to manage their lives with the disease through the careful application of insulin and the control of the disease at the workplace. Every issue of the journal contained one article on diabetic education dealing with an important facet of diabetes management. Oliver Falk (see endnote 2) has argued that physicians’ support for patient self-care management was also motivated by acquiring knowledge about patient behavior for insulin standardization. But even if they exploited patient experiences for the reason mentioned, the patient-physician relationship was not a one-sided one belonging to a traditional medical hegemonial treatment regime. As we will see, the initative by Beining and his collaborators contributed to broadening the scope of diabetic patient management. It enabled patients to use their acquired freedom in diabetes self-care to rearrange those aspects of their lives which were affected by their disease: Since the editorial board consisted of laypeople as well as physicians, the journal reflected this dynamic and enabled the exchange of different opinions on diabetic care and problems related to diabetes between medical experts and laypeople.^8^

From its early days, Robert Beining and the Diabetic Association agitated against the idea that a diagnosis of diabetes meant continuous starvation. Combining dietary measures together with the application of insulin could now enable patients to lead an ordinary life with an almost normal life expectancy. By being informed and disciplined, the patient could manage his work just as healthy colleagues did. In light of this improved management, Beining held, it was wrong to declare diabetic patients unfit for work in an early stage of their career. In April 1956, Richard Boll, the district-chairman of the DDB Hamburg, pointed out in his speech on “the social duties of the German Diabetes Association” that “educated and disciplined diabetics have no major non-productive time compared with other men. Also, they need not to be declared disabled prematurely.”^9^ This modern vision had been delivered by the German Diabetologist Gerhard Katsch (1887–1961), who had since the 1930s propagated the idea of the diabetic patient as being “conditionally healthy.” Diabetes patients could lead ordinary lives when they kept in contact with their physician and adhered to diet and insulin application recommendations. In fact, employment represented one cornerstone of Katsch’s scheme, since it lent patients self-affirmation and social security (Prüll 2012: 31–61; Knick 1968; Falk 2019: 170–174).

Because this attitude did not correspond with the public image of the diabetic patient, the job situation of diabetes patients remained precarious, especially in the case of civil servants. In 1956, a 26-year-old patient sent a letter of complaint to the journal *The Diabetic*. His transfer to a civil servant position was refused by the administration of a German town since “early disability has to be envisaged because of diabetes.” The patient now asked for help and especially for an acknowledgement of the new perspectives on the diabetic working life:

… many colleagues are already taken over into civil service […] I would like to ask you, whether—from your point of view—there are really no options to become a civil servant as diabetic. Aren’t attitudes about early disability of diabetics outdated? Do you know, how these matters are handled elsewhere? What would be your further advice?^10^

This patient—one of many—was not only concerned about adequate dietary measures and the accurate insulin dose. At stake was his work life and his working place. So he took the initiative to change his labor conditions.

News of improved treatment management fueled the debate on discrimination against diabetic patients. In 1957, the chairman of the DDB district group in Düsseldorf, the physician Paul Schwenger, aggressively advocated for the right of diabetic patients to work and to be accepted as regular workers. Schwenger’s speech received significant attention since it was held on the occasion of the foundation of the Düsseldorf Group. Schwenger promised that he could “offer the Ministry of Labor a reserve work force of 200,000 to 300,000 out of the community of diabetic citizens.”^11^ In the very same year, the diabetes specialist Rudolf Pannhorst (1904–1983), leading physician of the district hospital Gelnhausen, complained that a diabetic would never get a civil servant position, although there already were some who were qualified. He pointed out that if a diabetic patient was known to be compliant with treatment and if occupational risk factors were low, these individuals could be expected to work normally and have an average life expectancy (Pannhorst 1957: 163)^18272^. Reports from the United States affirmed this position: Diabetics would be completely able to fullfill duties as civil servant (Schweisheimer 1957). And in 1958, even case studies underlined these positive expectations regarding the suitability of people with diabetes for state service (Northoff 1958).

Although physicians played their part in promoting the productivity of diabetes patients, it was the layperson Robert Beining who launched the decisive initiative to improve the options of diabetic patients in the public service. In April 1958, he wrote a letter to the Federal Minister of Labor and Social Affairs.^12^ In it, he described how diabetic patients were excluded from civil servant positions although they were just as productive and effective as their healthy colleagues and enjoyed the same life expectancy. This was well-known in the USA, where since 1941 diabetes no longer prevented anyone from obtaining a governmental position.^13^ Beining based his arguments on the persistence of prejudices against diabetic patients, pointing out the changes to diabetes treatment since the introduction of insulin.^14^ He asked for a reassessment of the regulations for employment regarding diabetic patients, mentioning that almost 15,000 young carrier of diabetes would be excluded from work. With this argument, Beining attempted to demonstrate the potential of persons with the disease to fulfill their part in reconstructing West Germany. After 1950, the quota of unemployed workers in the country fell dramatically: In 1950, it was 11%, in 1955, 5.6% and in 1958, when Beining launched his initiatve, the quota was only 3.7% (Statista 2021). This trend indicated the need for workers in many areas and revealed the imperative to use workers as efficiently as possible. Additionally, Beining encouraged the Federal Minister of Labor to contact the Insuline Committee, a panel of diabetes specialists set up in 1948 to control and improve standards for the application of insulin. In doing so, Beining hinted at the support of therapists to push his interests through.^15^

At this point it is important to consider the background of diabetes patients’ problems with civil servant positions and the reasons for Beinings’s appeal. As “state servants” these civil servants had a special position, which had their origins in the eighteenth-century efforts of the local elite to build up a reliable staff for territorial administration. In these positions, they were bound to the sovereign for their whole lives. In exchange they also enjoyed special privileges, such as a life-long income and other advantages. After 1945, these posts became disreputable since they had been abused to pursue the racist policy of the Nazi regime. Nontheless, it was decided to keep up the civil servant system. Different laws in the German states were bound together in 1957 when the Federal Republic set out civil servants’ activities when creating a specific law, which assimilated regional specifities within a general guideline for administering civil servant posts (*Entstehung und Entwicklung des Beamtenrechts in Deutschland*, 2021). This “Framework Law to Unify Civil Servant Law” (*Rahmengesetz zur Vereinheitlichung des Beamtenrechts/Beamtenrechtsrahmengesetz—BRRG*) stipulated that “Appointments should be made on the basis of aptitude, ability and professional competence without consideration of sex, origin, race, believe, religious or political attitudes, origin or personal relationships.”^16^ Admittance to the civil service required a medical examination, which had to be carried out by health officers of the local health authorities as representatives of the state. Therefore, Beining directed his request to the Federal Ministry of Labor and Social Affairs without involving any regional governmental bodies.

The Ministry mentioned received Beining’s letter (Department No. I) and did not react for a month. Remarkably, in May 1958, the matter then was delegated to the Department No. II, which was in charge of care for “disabled persons.” This delegation illustrates the image of diabetic patients as disabled that still prevailed. Nonethless, Beining’s request was taken seriously. Plans were made to evaluate the matter and to include the Federal Ministry of Internal Affairs for further decisionmaking.^17^ The Federal Ministry of Labor sent a request to all major federal agencies, asking to gather reports of experiences with young diabetic patients. But the reaction was weak. The German Guild of Craftsmen (*Deutscher Handwerkskammertag*) sent a reply with the comment that they had no data regarding the problem. Also, the German Industry and Trade Company (*Deutscher Industrie- und Handelstag*) mentioned that they had “no material.”^18^ Therefore, the Federal Ministry of Labor and Social Affairs could not develop an effective plan to cope with diabetic patients.

In the course of July 1958, the matter was handed over to the Federal Ministry of Internal Affairs, which at the time acted as a department the Federal Health Office (*Bundesgesundheitsamt*).^19^ The president of the latter produced a detailed report on the situation of diabetics on the job market in light of recent standards of medical care. Although there was no statistical basis, the president agreed with the German Diabetic Association that diabetic patients “mostly and regularly were open-minded, intelligent people, ready for service.”^20^ On the one hand, he argued that diabetic patients could work in the field of administration. On the other hand, he had only weak statistical information from regional health insurance agencies and argued, therefore, that Beinings number of 15,000 young diabetic patients who were fit for work represented an overestimate. Furthermore, information about early retirement for health reasons would be missing and every single case should be investigated independently. Finally, therefore, the president of the Federal Health Office refused to pursue further measures regarding this matter.^21^

Nonetheless, diabetologists backed the attempt of the Diabetic Association to have the issue addressed. Increasingly diabetes management was viewed in line with Gerhard Katsch’s views as a psychosomatic and holistic process that required collaboration with the patient as well as her integration into the labor world. Although Katsch worked at the University of Greifswald within East Germany, his voice was still heard in the west. He was still active until his death in 1961 shortly before the erection of “the wall” (Falk 2019: 174–176; Prüll 2013: 231–232; Berger et al. 1990: 80–84). In 1958, the diabetes specialist C.H. Mellinghoff estimated the number of diabetics in West Germany at 500,000 and Paul Schwenger, repeating and confirming his earlier remarks, declared this number to be an unemployed workforce that should be mobilized in collaboration with the state offices for labor. Similar to Beining, Schwenger alluded to the recovery of the West German economy and the reestablishment of society, which rested upon achieving economic success and some degree of private luxury.^22^ Additionally, the Insulin Committee approached the Federal Ministry of Internal Affairs on February 6, 1959 to report the same information as Beining did, thereby reinforcing the request of the Diabetic Association with medical expertise. The committee focused on the outdated prejudices against diabetes patients and pointed out that the introduction of insulin marked a turning point in diabetes therapy. The committee additionally added a draft of new guidelines for the appointment of diabetes patients to civil service positions and instigated further discussions on the subject.^23^ These guidelines delivered ten points for medical expert employment advice, listed below:

1. Based on medical, economical and labor-law related reasons, a general exclusion of diabetic patients from employments with claims for pensions in governmental posts or equivalent positions can no longer be justified.
2. All diabetic patients capable of work whose metabolic disorder can be adjusted with or without insulin and who are seemingly capable to work until regular retirement age can be employed.
3. Applicants must have no essential diabetic complications, especially those related to the retina or the kidneys.
4. Applicants must submit medical evidence testifying to a good metabolic condition as well as sufficient control of blood sugar. Diabetic patients without control of blood sugar pose a risk for employment and should be refused.
5. Evaluation of the “good adjustment” depends on blood and urine data and to a certain extent on the insulin dose. These should be performed individually. Generally, a diabetic patient is “well adjusted” with fasting blood sugar under 180% and 3 hours after meal under 250%, within urine not above 10 g (in case of daily carbohydrate intake of 150 g), and in case of increased carbohydrate intake not more than 20 g per day. There should be no disposition to cetonuria.
6. Admittance to a civil service post should take place only if the applicant is over 25 years old and her diabetes is under permanent control for at least two years. Hiring on probation for one to two years with final evaluation should be performed in cases with low risk but not enough evidence.
7. Applicants with diabetes fulfilling the prerequisites mentioned above should be admitted to the pension fund without any further limitations. Usually, the fitness for employment should be clarified individually by expert medical assessment.
8. Diabetic patients with the necessary education and knowledge, who need no insulin, can carry out the respective job like someone else.
  Diabetic patients who need insulin should avoid jobs with irregular working hours (change of shifts, night work between 24:00 and 7:00) to avoid changes to insulin and dietary plans. Also—for reasons of security—they should not be committed to activities which endanger themselves and others in case of hypoglycemia (work on scaffolds, using machines etc.).
9. Every diabetic patient should be examined in intervals, which should be determined on a case-by-case basis. Generally, these intervals should not exceed 12 weeks. Every year, a thorough general medical examination should be carried out.
10. In case of complications, which do not restrict the working capability of the respective person but suggest the change of the working place, this change should be arranged by the medical health officer responsible.^24^

In summary, these guidelines represented a compromise between the needs of governmental institutions to secure the employment of healthy candidates and the right of diabetes patients to be acknowledged as candidates who are able to fulflil the demands of civil service. The role of the medical expert and his authority was embedded in this scheme, yet the medical expert also acted as a spokesman of patient rights. In conclusion, he served as a mediator between the state and the patient.

The combined initiative of the DDB and physicians exerted pressure on the Ministry of Internal Affairs. The latter agreed—with only minor amendments—to the new guidelines for employment of diabetic patients as a recommendation for future use in almost all ministries of the Federal Republic. These guidelines provided the basis for the employment of diabetic patients who where at least 25 years old and had their disease under medical control at least for two years and with no long-term damages. The adoption of these guidelines represented a historic break, because now diabetic patients could be appointed as civil servants.^25^

## Reshaping the Qualification of the Diabetic—New Discussions After 1969

Although successful, the guidelines did not solve problems in the long run. They remained too general. Prejudices surrounding the employment of diabetic patients could be revived if necessary. Therefore, critics called for amendments and changes. Eleven years later, in 1969, one specific event promoted new discussions about the guidelines. Its starting point were the activities of Hellmut Mehnert (*1928), a diabetes specialist and medical pioneer in diabetes treatment in Munich. Mehnert had worked as a guest physician in the USA, where he had collaborated with Elliott P. Joslin. In 1969, Mehnert was appointed director of the Third Medical Department of the Clinic Munich-Schwabing. Later, he became a nationally and internationally well-known physician and scientist, who stood for patient-adjusted medical practice.^26^

In 1969, Mehnert was asked to evaluate the status of an applicant for a civil servant position at the Federal Postal Service. The Postal Service administration urged Mehnert to perform specific food tests and above all to determine the fasting value of blood sugar. Mehnert was embarrassed by the request and sent a complaint to the administration, on the basis that the tests were outdated and they would endanger the patient’s good health based on a well-belanced blood glucose value.^27^ An exchange followed about the nature and the extent of the examinations necessary for decision-making. The Federal Ministry of Youth, Family and Health addressed the Insulin Committee with the request to deliver a comment on Helmut Mehnert’s complaint. Since the Insulin Committee had been taken over by the German Diabetes Society (*Deutsche Diabetes Gesellschaft*, DDG) as the preeminent body of diabetes specialists,^28^ the request was handed over to the chair of the society’s Panel for Social Medicine, headed by Günther Kurow (1921–2001). Kurow was to send a statement to the Ministry.^29^

Kurow was the perfect choice to deal with this matter. He himself was diabetic, which gave him insights into the living conditions of his patients. Furthermore, as an occupational physician, he was well informed of workplace problems facing diabetic patients. In Berlin, where he had his own medical practice, he had streamlined medical diagnostics and therapy of diabetes patients. At the same time, he was engaged in social work, such as the founding the Berlin Diabetic Social Services (*Berliner Diabetiker-Sozialwerk*) (Kurow 1996: 9, 46–51, 58/59; 1961: 23–27).

After Mehnert had launched his complaint, similar messages were sent off by diabetic patients, who contacted the journal *The Diabetic*. In June 1970, the story of an employee of an insurance company (*Allgemeine Ortskrankenkasse, AOK*) was published. The employee’s application for public servant appointment had been rejected by Public Health Officers and only a second medical reference based on the support of the German Diabetic Association had enabled the employee to achieve the position wanted (Beamtenernennung trotz Diabetes 1970: 23). Two months later, the journal published a second report. This time, a 21-year-old police man was dismissed from his job as he was found unfit for service in the riot police. Then his application for the civil service was rejected because public guidelines for employment of diabetic patients for public service would allow employment after the age of 25 only in exceptional cases. A representative of the government agency told him “you can come back in a few years.” The journal commented that often in such cases applications or appointments would be “rejected by administrative bodies for flimsy reasons.” Diabetic readers continue to deliver stories of discrimination and unawareness of medical personnel.^30^ In the same year, the German Diabetes Association sent an open letter to the chancellor of the Federal Republic of Germany with a complaint regarding the situation (España Munðz 2020: 33).

Günther Kurow took up the challenge to improve the fate of the so called “diabetics.” Beginning in 1970, he forcefully tried to improve the guidelines from 1958 by looking at different employment options.^31^ Finally, diabetologists decided in favor of new regulations in 1971. The new regulations had no age restriction for applicants and clarified the method for measuring blood and urine values. Also, a more detailed description of jobs unsuitable for diabetes patients was given (§ 8): “for example driver of public traffic vehicles, driving and handling of powerful machines, working on scaffolds. This is also true for diabetics with a tendency for hypoglycemia, receiving a high potency treatment with oral antidiabetics.”^32^ Although these changes were intended to provide relief to patients, the change of §4 also strengthened the position of medical experts:

4. Applicants must submit medical evidence testifying to a good metabolic condition as well as **regular and successive** control of blood sugar **and their cooperativeness**. Diabetic patients not controlled sufficiently **and being inccoperative** pose a risk for employment and they should be refused. **The employability of the applicant as a general rule should be checked by medical expert reference.**^33^

This means that the clarifications and improvements for patients were accompanied by loss of self-management. The price the “diabetics” paid was a more rigorous medical observation with regular medical examinations. Specific activities such as driving machines in public were excluded as job options and there was a more subtle differentiation between insulin dependent diabetic patients and older ones, who merely required dietary measures. Most remarkably, all patients deemed “not cooperative” (*unkooperativ*) were excluded from possible governmental service. Patients had the obligation to maintain their health status in regular consultation with their diabetes specialist.^34^

In 1971, the Ministry of Youth, Family and Health accepted the new guidelines and recommended its application to the other federal institutions.^35^ The guidelines represented a compromise between patients’ and physicians’ interests, brought forward by Kurow, who could play the role of a mediator between both groups. But problems with the implementation continued, since institutions sometimes had contradicting standards regarding the handling of medical evidence and the evaluation of examinations. In 1972, the public health office of Bruchsal refused the tenure appointment of a teacher despite the fact that a specialist of internal medicine as well as the ophthalmological university hospital of Heidelberg had confirmed that the person was in good health.^36^

Therefore, discussions about the guidelines did not cease. In conclusion, two key aspects that fueled the continuous discrimination of diabetes patients can be identified. The first one was the question of diabetes patients’ status as “severely disabled” on the basis of a certain certificate which allowed them to apply to public servant posts successfully. Since it was more convenient to have the certificate than to quarrel and discuss with employers, diabetes patients often decided in favor of this document. Since about 1976, the new diabetes guidelines were successively hampered by the disability option. In accepting the certificate, diabetic patients agreed to their own pathologization in order to secure a job. Günther Kurow tried to convince his fellow diabetics to resist taking this step. In his view, it torpedoed all the efforts of the *DDB* and the diabetes specialists within the *DDG* to describe diabetic patients as “conditionally healthy” (*bedingt gesund*). He therefore argued at the ministerial level against this “Danaian gift” (*Danaer Geschenk*). Kurow argued that it would be possible to adjust treatment effectively to avoid complications and thus to enable state service. The status of “severe disability,” in contrast, hampered all legislative efforts to grant diabetics protection, since it led to a general, fundamental discrimination against them. Kurow supported his statement with the attachement of patient files.^37^

The second key problem associated with the guidelines concerned state representatives within the healthcare system. It became evident during the late 1970s that the attitude of the health officers (*Amtsärzte*) was one important hindrance to improve diabetic patients’ rights regarding civil servant appointments. This group acted as the “gate keepers” to jobs in governmental health institutions, backed by a long tradition of state-supported conservative policy. They proved to be a decisive backbone of public health policy during the Nazi period as they were adherents of a segregationist racial hygienist agenda (Labisch & Tennstedt 1985). After 1945, American Occupational Forces tried hard to instill the values of the new democracy in the West German health care system (Ellerbrock 2004). Although the health officers collaborated in the reconstruction of health infrastructure, many did not change their basic attitudes. At the beginning of the 1950s, the health officers refused the compensation claims of diabetic patients for adverse effects of wartime on proper treatment, arguing that diabetes was a “hereditary constitutional insufficiency of the pancreas,” based on a “hereditary defect” (*Erbfehler*) in the family of the patient. Health officers continued to use this justification, even when the diabetic patient was the only one in the family with no hints of any hereditary origins of the disease. The request for compensation was rejected with the remark that hereditary laws would be too complicated for laypeople to understand and that there would be latent (hidden) hereditary defects (Sido 1951: 40). This last phrase was in fact a “knockout argument,” alleging hereditary damage without any prove of evidence.^38^

Based on these racial hygienist thoughts, many health officers interpreted the guidelines in their own way. To a certain extent, they abused them to prevent the employment of young diabetic patients as public servants. During the 1970s this led to a conflict between patients and health officers. Diabetologists tended to support their patients. The conflict between patients and diabetologists on one side and health officers on the other side finally escalated in 1978. The diabetologist Joachim Kühnau visited the sessions of the Panel of Medical Assessment of the Committee of Excecutive Health Officers (*Ausschuss für das ärztliche Begutachtungswesen der Arbeitsgemeinschaft der Leitenden Medizinalbeamten, AGLMB*) and tried to convince his health officer colleagues that a diabetic patient with finely adjusted insulin dosage and dietary measures could do her job over the course of three or four decades without problems. Kühnau further argued that persons with diabetes would behave extremely responsibly; he used traffic accident statistics to make his point.^39^ In the course of the debates, it became clear that the Health Officer Panel had argued with numbers from 1950–1952 when asserting that diabetes patients would have only a life expectancy of 19.5 years in case of an outbreak of the disease in the second decade of life.^40^ In the final discussion, the Health Officers agreed to the improvements made in health management for young patients. Although they ultimately did not revise their evaluation guidelines for applicants with diabetes, they recommended that Kühnau should further contribute to the guidelines of the *DDG* to give more details about criteria for estimating adequate healthcare in young diabetic patients.^41^

In parallel, the ministries became sensitized regarding the conflict. In 1978, Günther Kurow wrote a letter to the Federal Ministry of Youth, which included remarks about the attitudes of the Health Officers:

You described the tendency of Health Officers to use the guidelines of the German Diabetic Association to justify the rejection of applications with the argument that one could not expect a regular lifespan of work. This leads to a lopsided discrimination against diabetic applicants within the group of severely disabled persons.^42^

In the following, Kurow proceeded with his activities in favor of civil servant employments of diabetic patients. He pointed out their conditional health and the discrimination they faced when compared with other severely disabled applicants. Kurow backed his initiative with strong evidence: In April 1977 he had started an appeal in the *Diabetic Journal *asking diabetic patients to send him commentaries about their lives and their problems obtaining a civil servant position. He chose sixteen patient letters and added them to his own commentaries.^43^ This way, the individual stories of difficult job searches and the discrimination against diabetic patients in everyday life became visible and comprehensible.

The debates of the following decades are not the topic of this paper, but they should be summarized at least: The initiatives described were successful in changing the spirit and the attitude within the debate, which in the future contributed to the usage of the guidelines to employ diabetic patients. Although from 1970 onward the unemployment quota was rising (from 3.8% in 1980 to 9.3% in 1985) (Statista 2021), discussions about the job market were not decisive in the arguments advanced by Kurow and others; instead, they argued in terms of intrinsic patient rights. However, the often-precarious job situation of this patient group remained. It is often a fact of life for many patients suffering from chronic disease. Even today, the employment of diabetic patients still depends on a medical examination, whose interpretation is based on the attitude of the examiner. At least, on the basis of a court trial from 2013, the evaluation of the prognosis of the disease has changed considerably: Until then, an inability to work had to be excluded with “a high degree of probability.” Since the 2013 case, there need to be “clear indications” that inability to work before reaching the pensionable age will occur “with overwhelming probability” (Schulze 2014; DGB Rechtsschutz GmbH 2021). Although this decision represented another step for removing hurdles to becoming a civil servant, the discrimination against diabetic patients persists even today. Kurow’s attempt to discourage diabetes patients from applying for a severe disability passport to obtain civil servant employment failed: Today, the chances of a diabetes patient successfully securing employment are still far better with the label of being “severely disabled.” Although medical advice was not successful in this case, it nonetheless shows how patients themselves influenced disease management—even on the job market: Today it is widely acknowledged that diabetes does not lead to an inability to work efficiently in civil service. Furthermore, all the discussions after 1969 carried on the process of a more subtle and careful investigation of the individual’s health and fitness, which can be seen on display in recent court trials regarding the question of civil service employment (Diabetes & Recht 2019; Diabetes 2019).

## Diabetes, Self-Expression and Patient Rights

After 1945, diabetic patients were confronted with old prejudices even in the democratic society of West Germany. The challenge for these patients was to improve their situation. Patients, lay supporters, and physicians could decipher outdated views on the disease and translate modern diagnostic and therapeutic values. Together with their supporters, diabetic patients constructed a new view of the disease, creating the image of a patient who was no longer a victim of starvation, but instead a “conditionally healthy” person with average life expectancy. This change of perspective extended access to diverse domains of social life for diabetics.

This story of empowerment overlaps with the history of other patient organizations in Germany after 1945. Though every organization worked in a unique context that strongly depended on the disease or problem, there are certain commonalities that can be observed (see Söderfeldt 2012; 2020).^44^ The job market remains an important sphere of activity; this is above all true for diabetes patients in discussions about their options in achieving a civil servant position. In 1958, the lay supporter Robert Beining mobilized diabetes specialists to translate the new meaning of diabetes for politicians and for the official health administration to advance the interests of diabetic patients. He was the first “mediator” who raised awareness about the diabetic question among public officials. The term “mediator” refers to the idea that individuals are members of different social groups, enabling the existence of boundary work, boundary objects, boundary knowledge and to a certain extent “boundary persons” (Söderfeldt 2020: 94). Beining was no diabetic, but nonetheless played an important role in helping diabetes patients. He was also a journalist who knew the ropes of public communication and used this expertise to tackle the problem of diabetic patients on the job market and especially their prospects for becoming a civil servant. Following therapeutic regimens that were widely available after 1950, diabetes patients should have enjoyed the right to apply for posts as public civil servants (*Beamte*). Beining’s initiative was successful, because the guidelines set up by the Insulin Committee of Diabetologists were accepted by diverse West German ministries. He was the first mediator within our context, who brought the intitiative for diabetic patients one step forward.

However, diabetes patients had options in principle, but not necessarily in practice. Complaints created a tensed atmosphere. Desperate for job perspectives, diabetes carriers sought to improve their chances by obtaining status as “severely disabled” persons. Whereas in 1958 lay helpers as Beining and diabetic patients had been the key figures who started the campaign for the civil servant option, by 1970 diabetologists had assumed a key role: twelve years later, these diabetes specialists argued with the ministries against the pathologization of patients as “severely disabled.” Physicians, such as Helmut Mehnert, whose education allowed them to act as “mediators,” advanced this argument. Mehnert mobilized knowledge acquired in the USA, where professional diabetes treatment had started, to counter prejudices. The second decisive person was Günther Kurow, who as diabetic patient himself had access to the experiences of diabetic patients. Both men translated and communicated ideas between the diabetic world and their fellow citizens within West German society. A similar story played out in other contexts: for example, in London the physician Robert Daniel Lawrence (1892–1968), also a diabetes patient, had widened the scope of his biochemical work into the sphere of diabetes treatment, thereby pioneering the foundation of patient self-help groups. Similar to Kurow’s later work, Lawrence had tried to mobilize diabetic patients to improve their situation when networking in society and politics (Prüll 2005: 1367f.). Just like Lawrence, who was also admired in Germany,^45^ Kurow mobilized diabetic patients and the ministries against conservative health officers, who seemed to hamper all successes made so far.

Mehnert, Kurow, and Lawrence in Britain were advocates of diabetes patients in matters of lifestyle. On the other hand, they never gave up their ideas about professional medical conduct. In order to improve the guidelines of 1959 and the prospects for employment, they needed to promote the idea of medical control of diabetes patients. Through this mediator work, both patients and physicians benefited from their collaborative translation of knowledge about diabetes. Diabetic patients gained a foothold in the sphere of civil service, while diabetes specialists extended their interpretative role and consolidated their influence as indispensable therapists and consultants. Collaboration between patients and physicians involved more than simply granting patients freedom to perform their own medical insulin dosage research.

The successful attempts to fight against the discrimination of diabetic patients promoted by the likes of Beining, Mehnert and Kurow was accompagnied by other initiatives, which also aimed at the advanced integration of diabetes patients into societal life. Therefore, the story of the diabetes patients’ struggle to obtain civil servant positions appears as the final chapter of this story. First attempts to integrate this group into social life involved the initiative of patients to improve their living conditions related to marriage and sexual life. During the second half of the 1950s, diabetic patients forced a discussion about intimacy and sexuality on their physicians, who often claimed marriage between diabetes patients and healthy citizens would result in diabetic offspring. According to the prejudices of the health officers, even diabetes specialists had concerns regarding hereditary origins of diabetes. Therefore, they tried to convince their patients to avoid marriage with diabetic patients. But the latter resisted and forced the editors of the diabetes journal to promote marriage and engagement advertising. This way they could increase their chances of partnership in spite of the burdens of their disease. These processes were also based on negotiations and discussions between diabetes patients and diabetologists, who needed to be convinced to follow the ideas of their patients: diabetic patients advocated for the acceptance of their own needs and attitudes. As early as 1956, the taboo subject of sexuality was brought out into the open. Through patients’ initiatives, which were soon embraced by diabetologists, they changed both the treatment and the therapeutic concepts of the disease itself. This process in turn promoted the democratization of medicine, as the empowerment of the patient was accompanied by other similar processes in the first decades of the Federal Republic. (Prüll 2012).

The second project taken up by patients was related to diabetes therapy itself, namely dietary measures. It involved diabetic patients redefining their “sense of life” as an expression of growing self-esteem; in many cases they cultivated their dietetic life as a counterpoint to hedonistic glutony of West German society in the 1950s. Based on the sense-of-life theory of the philosopher Paul Tiedemann, who focused on self-awareness as a cornerstone of a satisfying life experience, it was possible to analyze the reaction of diabetes patients to the increasingly well-to-do lifestyle in West Germany: Diabetes patients praised the resistance to the temptation of luxury foods, smoking and drinking. Moreover, they held themselves up as “ideal” members of society. One diabetic stated in 1957: “We do not live for eating, but we choose our food in a way to be able to fulfill our duties to society and to the family we have founded” (Prüll 2013: esp. 233).

The matter of civil service employment forms a third part of this history, since the patients together with their helpers and advocates remolded the image of the disease diabetes. Achieving the path to civil service employment was another step on the way to integrating diabetic persons into the job market and work life. Although employment guidelines came out of compromise with diabetologists, the improvement of employment prospects for diabetic patients as civil servants can be understood as emblematic of a significant development in patient rights and a departure from the long shadow of nineteenth-century paternalistic medicine and the excesses of Nazi Germany.

## Footnotes

1 Another example are transident/transsexual persons, whose fight for depathologization since about the 1990s involved explaining their own identity and their own scope of life to physicians as well as society: Prüll (2016: 265–293).

2 This is also true in the case of diabetes: Based on approaches to science theory, Oliver Falk reduces the problem of therapeutic responsibility and therapeutic action to the paternalistic top-down decision making of medical specialists, who used patient freedom to extract knowledge useful for the standardization of insulin. The patient’s role in this setting is the one of subaltern recipient of orders. In contrast, it is important to acknowledge that the need to acquire knowledge on insulin impact and effect is only one aspect of the rearrangement of the physician-patient relationship. The latter is also molded and influenced by autonomous patient acitivtiy and contemporary political conditions. This is true especially for the research period of this paper, namely from 1950 to 1970. See Falk (2018: 39, 54–56).

3 In the case of diabetics, the patients themselves were keen to improve their own nutrition, since their physicians made only meager efforts to teach and explain matters on this area. Uptake of diabetes therapy after the introduction of insulie therefore could be realized only on the basis of active cooperation and collaboration of patients and not only on the bases of a passive one. See again Falk (2018), but also Pfaff (2019) and Söderfeldt (2020: 30–42).

4 See the papers already published by the author, quoted and described in the last chapter of this contribution. Furthermeore, see the very short and rough description of the civil servant problem of diabetics by: Isabelle España Munðz, *Das Ärzt_innen-Patient_innen-Verhältnis von Diabeteskranken und Diabetolog_innen anhand des Diabetes Journals in der Bundesrepublik Deutschland zwischen 1970 und 1990*, Diss. Univ. Mainz (2020: 33–36).

5 Oliver Falk argues that uncertainty about types of diabetes during the 1920s and 1930s would have prevented knowledge about the shift of the diseases character from a short-term and deadly one to a long-lasting and chronical disease. However, this does not hold up to scrutiny since the basic effect of insulin on prolonging the life of patients became visible quite soon, leading even to the set-up of self-help groups already during the 1920s. See Falk (2018: 42); furthermore Prüll (2005: 1367f). For the history of diabetes with a focus on Germany, see also: Prüll (2014).

6 This was a sort of follow up to an earlier foundation of 1931 by E.O. Erdmenger in Berlin, which was disbanded by National Socialists in 1934. The journal of this first association, founded also in 1931, was entitled “Wir Zuckerkranken.” See Roth (1993: 3–10); Brettschneider (2001: 7).

7 The commission was headed by Dr.phil. Erich Both, director of the sanatorium at Bad Neuenahr. Both worked since 1945 in Bad Neuenahr and made his way to the head of the institution. He had been assistant of the internal physician Paul Martini (1889–1964) in Bonn, who was the attending physician (Leibarzt) of the first West German chancellor Konrad Adenauer and one of most influential physicians of postwar West Germany. See Beining (1951: 69/70). Erich Both was at least able to win Martini over to support the Association in non-material ways: Both (1964: 283).

8 In 1952, one of the founders of the journal, the missonary Hans Ziegler, criticized the poor readibility of the medical articles. In January 1953, the head of the medical commission of the journal, Erich Both, repeated the comment that the reader should be expected to work his way through more demanding articles. Ziegler (1952: 58); Both (1953: 1). Since the German Diabetic Association has no archive, it is not possible to follow up the origin of the articles as well as the selection of the letters from readers.

9 Report on the meeting of the DDB District Group Hamburg (1956: 100). All translations by the author.

10 “Beamtenlaufbahn für Diabetiker ausgeschlossen? Leserbrief K‑H A,” *Der Diabetiker* 6 (1956), 5, 220.

11 Schwenger quoted in: Krapp (1957: 99) (translation by the author). Krapp delivered a short report on Schwenger’s remarks.

18272 Rudolf Pannhorst was an adherent of National Socialism and wrote about hereditary aspects of diabetes in the journal „Zeitschrift für menschliche Vererbungs- und Konstitutionslehre“, Vol. 25, 1941/42. Nevertheless, after 1945, he could achieve the postition of the leading as well as internal medic at the mentioned hospital in Gelnhausen and published again on diabetes and heredity at least until the early 1950ties. See: Kürschners Deutscher Gelehrtenkalender 1954; Ewert, Ewert & Boettiger 2014.

12 Robert Beining to the Minister of Labor, Würzburg, 18.4.1958, in: B 149. Bundesministerium für Arbeit und Sozialordnung, No. 6476, Rehabilitation.—Diabetiker: Beschäftigung im öffentlichen Dienst < no pagination >, Bundesarchiv Koblenz.

13 Ibid., p. 1.

14 Ibid., p. 1/2.

15 Ibid., p. 2.

16 “§ 7 Ernennungen sind nach Eignung, Befähigung und fachlicher Leistung ohne Rücksicht auf Geschlecht, Abstammung, Rasse, Glauben, religiöse oder politische Anschauungen, Herkunft oder Beziehungen vorzunehmen”, in: Rahmengesetz zur Vereinheitlichung des Beamtenrechts (Beamtenrechtsrahmengesetz—BRRG, vom 1. Juli 1957), in: Bundesgesetzblatt Teil I, Nr. 29 vom 04.07.1957, p. 667–691, here p. 669, in: https://www.bgbl.de/xaver/bgbl/start.xav?startbk=Bundesanzeiger_BGBl&jumpTo=bgbl157s0667.pdf#__bgbl__%2F%2F*%5B%40attr_id%3D%27bgbl157s0667.pdf%27%5D__1625670663654; < access July 9, 2021 >

17 Ministry of Labor and Social Affairs, Department I, Remark, Bonn, May 6, 1958; Department II, Notice, Bonn, May 14, 1958, two pages, in: B 149. Bundesministerium für Arbeit und Sozialordnung, No. 6476, Rehabilitation.—Diabetiker: Beschäftigung im öffentlichen Dienst. Bundesarchiv Koblenz.

18 Deutscher Handwerkskammertag to the Bundesminister für Arbeit und Sozialordnung, Bonn, June 13, 1958; Deutscher Industrie- und Handelstag to the Bundesminister für Arbeit und Sozialordnung, July 2, 1958, in: Ibid.

19 The German Ministry of Public Health (Bundesgesundheitsministerium) was founded only in 1961.

20 Der Präsident des Bundesgesundheitsamtes an den Bundesminister für Arbeit und Sozialordnung, Koblenz, July 11, 1958, in: B 149. Bundesministerium für Arbeit und Sozialordnung, No. 6476, Rehabilitation—Diabetiker, Beschäftigung im öffentlichen Dienst, 2.

21 Ibid., 2–8.

22 “500000 Arbeitskräfte könnten noch mobilisiert werden.” III. Internationaler Diabetikerkongreß in Düsseldorf, in: *Westdeutsche Allgemeine* v. 22.7.1958, in: ibid; Krapp (1957: 99).

23 Karl Oberdisse an den Bundesminister des Innern, betr. “Übernahme von Diabetikern in das Beamtenverhältnis”, Düsseldorf, 6.2.1959, in: B 106, Bundesministerium des Innern. Diabetiker, No. 107788; Unterhefter 210107/5 (Einstellung von Diabetikern in den öffentl. Dienst u. weitere Einstellungsvoraussetzungen 21. April 1958 bis 3. Oktober 1961, Heft 1), Bundesarchiv Koblenz.

24 Richtlinien für die Beschäftigung von Diabetikern, besonders als Beamte im öffentlichen Dienst. Deutsches Diabetes-Komitee. Arbeitsgemeinschaft der Deutschen Gesellschaft für Innere Medizin Düsseldorf, 6.2.1959 in: B 189/24265. Einstellung von Diabetikern in den öffentlichen Dienst; Bd. 1, Auseinandersetzung mit dem Bundesministerium des Innern über die Einstellung von Diabetikern im öffentlichen Dienst. Bundesarchiv Koblenz.

25 Der Bundesminister des Innern, 13.3.1959, in: B 189, Bundesministerium für Jugend, Familie, Frauen und Gesundheit (1969–1991) und Funktionsnachfolger, No. 24265, Einstellung von Diabetikern in den öffentlichen Dienst, Bd. 1, Auseinandersetzung mit dem Bundesministerium des Innern über die Einstellung von Diabetikern im öffentlichen Dienst. Anlage 1 des Schreibens: Bundesminister d. Innern (i. A. Dr. Braun) an den Bundesmin. f. Jugend, Familie und Gesundheit, Bonn, 19.12.1969. Bundesarchiv Koblenz.

26 See Mehnert’s Curriculum Vitae, in: https://www.diabetes-deutschland.de/mehnert.html; Interview with Hellmut Mehnert, done by the author of this paper on Nov. 11, 2010, Munich.

27 Prof. Dr. H. Mehnert, Städt. Krankenhaus München-Schwabing, III. Medizinische Abteilung, Chefarzt, to the Deutsche Bundespost, Oberpostdirektion, Munich, Sept. 23, 1969, in: B 189/24265 Einstellung von Diabetikern in den öffentlichen Dienst; Bd. 1, Auseinandersetzung mit dem Bundesministerium des Innern über die Einstellung von Diabetikern im öffentlichen Dienst. < no pagination >, Bundesarchiv Koblenz.

28 The German Diabetic Society was founded in 1964 (well after the foundation of the lay organization, the DDB) as a union of medical diabetes specialists. See: Berger et al. (1990: 17/18).

29 Bundesmin. für Jugend, Familie und Gesundheit to the Deutsches Diabetes-Komitee, Bonn, Feb. 19, 1970; Prof. Dr. H. Sauer, Secretary of the German Diabetic Association to the Bundesmin. für Jugend, Familie und Gesundheit, April 15, 1970, in: B 189/24265, Einstellung von Diabetikern in den öffentlichen Dienst, Bd. 1, Bundesarchiv Koblenz.

30 “Er darf kein Beamter werden,” *Der Diabetiker* 20 (1970), H.8, S. 29.

31 Aktennotiz, Bundesmin. für Jugend, Familie und Gesundheit, Bonn 20.11.1970, in: B189/24265. Einstellung von Diabetikern in den öffentlichen Dienst.

32 Richtlinien für die Beschäftigung von Diabetikern, besonders als Beamte im öffentlichen Dienst, 5.2.1971, in B 189/24265 Einstellung von Diabetikern in den öffentlichen Dienst; Bd. 1, Auseinandersetzung mit dem Bundesministerium des Innern über die Einstellung von Diabetikern im öffentlichen Dienst. Bundesarchiv Koblenz.

33 Ibid.

34 Ibid.

35 Bundesmin. für Jugend, Familie und Gesundheit, Bonn, an das Bundesmin. des Innern, 22.4.1971; Bundesministerium des Innern an die obersten Bundesbehörden, Bonn, 7.5.1971, in: ibid.

36 Deutscher Diabetiker Bund an Bundesmin. f. Jugend, Frankfurt, 21.1.1972; Oberschulamt Nordbaden an N. B., Hauptlehrer, Karlsruhe, 26.11.1971, p. 1–3, in: ibid.

37 Bundesmin. f. Jugend etc. an DDG, Ausschuß für Sozialmedizin, Bonn, Bad Godesberg, 15.7.1976; Günther Kurow an Irene Hohage, Bundesmin. f. Jugend, Berlin, 22.12.76; Günther Kurow an E. Hofmann, Kinder- und Poliklinik der TU München, Berlin, 15.11.1976, in: ibid.

38 The arguments of the health officers correspond with evidence on the massive ideologization of German society Early West Germany after 1945: See Herbert (2002: 15).

39 Digest of the Meeting of the Panel of Medical Assessment of the Committee of Excecutive Health Officers (*Ausschuss für das ärztliche Begutachtungswesen der Arbeitsgemeinschaft der Leitenden Medizinalbeamten, AGLMB*), October 12./13, 1978. Topic No.3, Civil Service Capability in case of diabetes, p. 9. in: B 189/24265 Einstellung von Diabetikern in den öffentlichen Dienst; Bd. 1, Auseinandersetzung mit dem Bundesministerium des Innern über die Einstellung von Diabetikern im öffentlichen Dienst. Bundesarchiv Koblenz.

40 Ibid., p. 7.

41 Ibid., p. 10.

42 Kurow to Dr. Loos, Federal Ministry of Youth, Berlin, 17.2.78, in: Ibid. < translation by the author >

43 Ibid.

44 Further research to elucidate the comparison between different patient self-help groups—such as that of Ylva Söderfeldt—and (international) comparisons of access to civil servant posts would expand the scope of this paper in useful directions. However, these further studies require additional methodological tools and a reframed paper lay-out. See the experiences of the author with comparative work and respective methodological remarks in: Prüll (2003: 26–30).

45 Interview with Helmut Mehnert, conducted by the author on Nov. 11, 2010, Munich.
